# Functionalized non-viral cationic vectors for effective siRNA induced cancer therapy

**DOI:** 10.1515/rnan-2017-0001

**Published:** 2017-05-27

**Authors:** Kshitij Gupta, Anu Puri, Bruce A. Shapiro

**Affiliations:** RNA Structure and Design Section, RNA Biology Laboratory, National Cancer Institute, National Institutes of Health, Frederick 21702, MD, USA

**Keywords:** RNAi, siRNAs, non-viral cationic vectors, cell penetrating peptides, lipids, polymers, functionalization

## Abstract

RNA interference (RNAi) has been regarded as a vital asset in the field of therapeutics as it has the capability to silence various disease causing genes including those that cause cancer. Small non-coding RNA molecules such as short interfering RNAs (siRNAs) are one of the extensively studied RNAi inducers for gene modulations. However, the delivery of RNAi inducers including siRNAs is compromised due to the barriers imposed by the biological system such as degradation by nucleases, rapid clearance, high anionic charge, immunogenicity and off-target effects. Viral vectors, in general exhibit high transfection efficiencies but are expensive and likely to confer immunological and safety issues. Therefore, non-viral cationic vectors (NVCVs) have received considerable attention to not only address these issues but also for developing efficacious siRNA delivery vectors. In this review, we will first discuss the historical development of various NVCVs and then will discuss functionalized NVCVs with linkers that provide stability, as well as respond to the cancer cell environment and with cancer cell receptor specific ligands to explicitly target them for improved siRNA efficacy. Multifunctional NVCVs (MNVCVs) that employ multiple synergistically working components to aid siRNA delivery efficacy are also discussed.

## 1 Introduction

**Figure 1.**
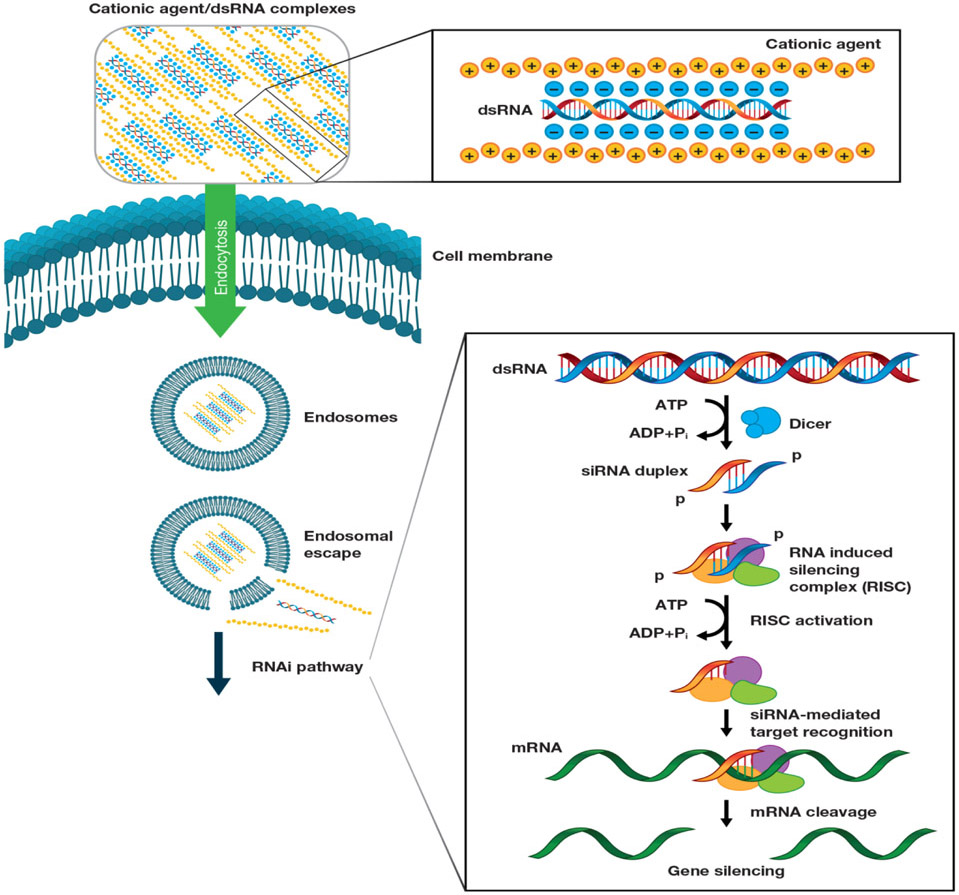
RNAi pathway. Schematic representation for silencing gene expression by siRNAs.

In recent years, RNA interference (RNAi) has emerged as a powerful tool for the study of gene function in mammalian cells [1]. In 1998, Fire and Mello first introduced this concept and showed that cell systems have the inherent ability to modify gene expression [2]. RNAi is a post-transcriptional gene silencing mechanism that can be triggered by RNAi inducers which include small non-coding RNA molecules such as short interfering RNAs (siRNAs), microRNAs (miRNAs), short hairpin RNAs (shRNAs) and piwi-interacting RNAs (piRNAs) [3]. Except for piRNAs, all other small non-coding RNAs are processed by the enzyme ‘dicer’ for further RNAi processing (Figure 1). However, they are all activated through the RNA-induced silencing complex (RISC) for gene silencing. The siRNAs, miRNAs, and shRNAs produce degradation and/or translational repression of the target when bound to mRNAs whereas piRNAs are involved in gene silencing by specifically silencing transposons [3]. For more detailed literature about small RNAs inducing RNAi, readers are requested to read the following references [1, 4, 5].

Inducing RNAi by small non-coding RNAs including siRNAs has become one of the critical assets for investigators not only to elucidate and classify pathways associated with cancer development and metastasis but also for use in the treatment of cancer. However, the therapeutic delivery of siRNAs is limited by several factors [6], (i) exo and endo-nuclease degradation which in turn results in a shorter half-life and fast kidney filtration [7], (ii) physicochemical features such as high molecular weight (~13 kDa) and high negative charge hinders its binding and crossing the cell membranes in comparison to small molecule entities, (iii) rapid uptake by mononuclear phagocytic system (MPS), (iv) immunogenicity, and (v) off-target effects [8].

To circumvent these limitations, investigators have applied several approaches for siRNA delivery. These include (i) chemical modifications of siRNAs (ii) utilization of viral vectors and (iii) use of cationic non-viral vectors. Chemical modifications of siRNAs can improve their stability in circulation; however, their efficacy is compromised due to off target effects and reduced therapeutic index [9]. Viral vectors are efficient delivery systems based on the use of genetically-modified viruses, exploiting their innate ability to introduce genetic material in eukaryotic cells [10]. However, these systems suffer from being immunogenic, having off-target effects and are expensive to produce [11, 12]. Therefore, efforts have been made in recent years to develop non-viral cationic vectors (NVCVs), since the cationic components have the inherent ability to electrostatically complex with the negatively charged siRNAs [13]. Initial efforts to develop NVCVs such as cell penetrating peptides (CPPs), lipids, and polymers show promise for intracellular delivery of siRNAs; however, they encounter limitations including cytotoxicity of the carrier itself and undesirable side and off-target effects [14]. These limitations led investigators to develop next-generation NVCVs that are functionalized with moieties that aid in cellular delivery, such as the cancer cell environment specific stimuli-responsive moieties and tissue-specific targeting ligands for improving RNAi efficacy [15]. In this review, we will focus on the historical development and current status of functionalization of NVCVs ranging from peptides to lipids to polymers in their respective sections. Since PEGylation is the most common functionalization for imparting stability *in vivo* to almost all the NVCVs [16], in this review we will not discuss PEGylation as a functionalization in detail. However, we briefly mention the properties of PEGylation in the cationic lipids section which can be applicable to other NVCVs if incorporated with a PEG molecule of choice. Multifunctional NVCVs (MNVCVs) that work seamlessly in synergistic coordination irrespective of distinct functionalities incorporated in a single carrier are also discussed. In the end, we will provide our perspective for the improvement of RNAi therapy using NVCVs.

## 2 Overview of NVCVs

### 2.1 Cell penetrating peptides

Cell penetrating peptides (CPPs) are a heterogeneous family of peptides with 5–30 amino acids that present great variety in terms of amino acid composition and 3D structure, with examples of cationic, anionic, and neutral sequences showing varying degrees of hydrophobicity and polarity. CPPs are generally derived from naturally occurring proteins of viruses and bacteria; however a few were synthesized after careful consideration of design principles [17, 18].

Ryser and his colleagues first reported that the cationic peptides such as poly-L-lysine can deliver drugs across biological membranes [19]. Serendipitously, it was discovered that HIV-1 Tat protein, when incubated with cells, trans-activates the HIV-1 LTR promoter [20]. Antennapedia homeodomain, a purified protein from Drosophila melanogaster, was also shown to enter nerve cells and exert transcriptional activity [21]. Later on, these proteins and their shorter peptide sequences were used for the delivery of drugs, proteins, nucleic acids and nanosystems to cells [22].

CPPs have been also intensively investigated to deliver nucleic acids including siRNAs for inducing RNAi in cancer cells [23]. The non-covalent electrostatic approach for the delivery of siRNAs has been preferred over siRNA-CPP conjugates due to cationic/ amphipathic CPPs and anionic siRNAs interactions in their natural form just by simple mixing at desired molar ratios for complex formation. However, conjugates suffer with the problem of irreproducible results, technical difficulties in synthesizing, poor yields, poor cellular uptake and risk of losing siRNA activity [24].

G. Divita and co-workers first reported the non-covalent vectorization of siRNAs with CPPs [25]. They showed that MPGΔ^NLS^ containing a hydrophobic domain derived from the fusion sequence of HIV gp41 and a serine mutated hydrophilic domain derived from the nuclear localization sequence of SV40 T-antigen efficiently vectorized siRNA and prompted significant knockdown of luciferase and glyceraldehyde 3-phosphate dehydrogenase (GAPDH) genes [25]. Subsequently, non-covalent approaches have been intensely studied for siRNA delivery and many excellent studies were published [23, 24, 26, 27] .

### 2.2 Cationic lipid based vectors

Lipid molecules have been sought-after as the primary components of vectors for drugs and pharmaceuticals [28-32]. Several features of lipids that make them attractive choice(s) include their biological nature, ability to assemble into discrete structures to accommodate payload of drugs [33], ability to manipulate particle size distribution [34, 35], and modification of their surfaces with specific moieties [30] etc. These basic traits of the lipid molecules have been exploited further for delivery of nucleic acids including DNA and RNA [36, 37].

Primary consideration for using lipids for nucleic acid delivery relies on the presence of one or more positive charges in the molecule (generally termed cationic lipids) that can electrostatically interact with negatively charged nucleic acids. It can be envisioned that electrostatic interactions play an important role in the generation of lipid-based nucleic acid complexes. Needless to say that the choice of lipid with desired hydrophobic, hydrophilic and linker domains will also dictate the resulting interactions with nucleic acids and overall particle design. In this section, we focus on the lipoplexes (liposomes), in the context of siRNA delivery [6, 14, 38, 39].

**Figure 2.**
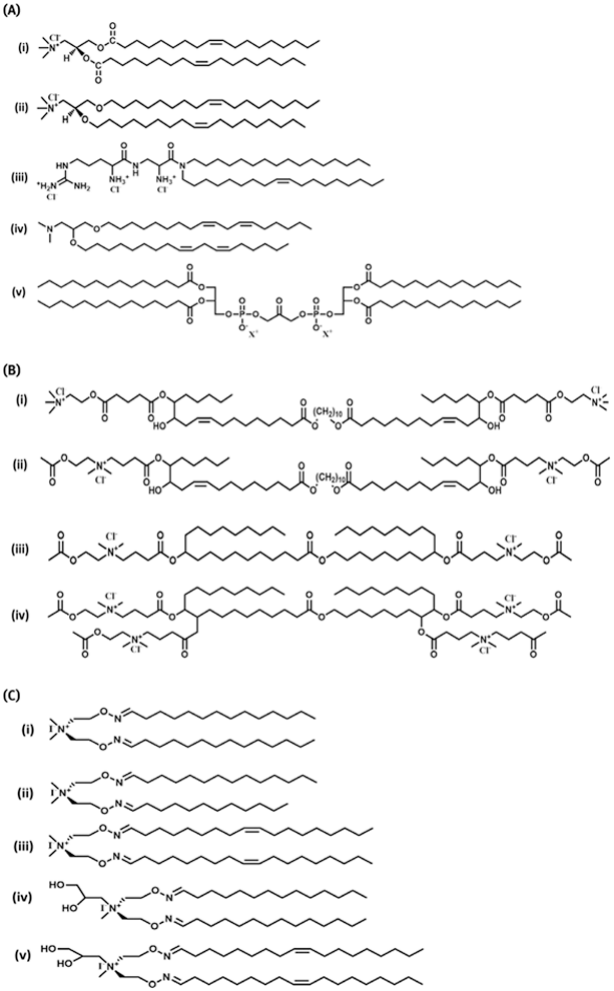
Chemically designed cationic synthetic lipids. (A) Extensively studied lipid molecules. (i) DOTAP, (ii) DOTMA, (iii) AtuFECT01, (iv) DLinDMA, (v) Cardiolipin analogs, (B) Head group modified bola lipids. (i) GLH-19, (ii) GLH-20, (iii) GLH-58, (iv) GLH-60, (C) Oxime ether lipids (i, ii, & iv) saturated; and (iii & v) unsaturated; (i, ii & iii) non-hydroxylated and (iv & v) hydroxylated head group.

In the field of nucleic acid (including siRNAs) delivery, two cationic lipids, 1,2-dioleoyl-3-trimethylammonium-propane chloride, (DOTAP) [40] and 1,2-di-O-octadecenyl-3-trimethylammonium propane chloride (DOTMA) [41] (Fig. 2A) have been extensively studied for cell culture-based systems [42] as well as for small animal studies [43, 44]. As expected, electrostatic interactions between the positive charges associated with the head groups of these lipid molecules and the negative charges on the siRNA are critical for the formation and stability of the resulting lipoplexes. It may be noted that the stereochemistry of the cationic lipids as well as their self-assembly characteristics also contribute to the overall efficiency of siRNAs transfection [45]. However, inconsistent transfection efficiency and cytotoxicity of these vectors prompted researchers to design new cationic lipids. Santel et al [36] wrote in the materials and methods section that they developed cationic liposomes comprising a novel cationic lipid containing three positive charges called AtuFECT01 (β-L-arginyl-2,3-L-diaminopropionic acid-N-palmityl-N-oleyl-amide trihydrochloride (Figure 2A), the helper lipid (see below), DPhyPE and the PEGylated lipid, N-(carbonyl-methoxy polyethyleneglycol-2000)-1,2-distraoryl-*sn*-glycero-3-phosphoethanolamine sodium salt (DSPE-PEG). AtuFECT01 liposomes showed significant silencing of genes in the vasculature of various organs.

In general, a lipid with a small cross-sectional area in the head group region and a larger acyl chain cross-sectional area exhibits a “cone” shape geometry that imparts H_II_ phase organization to enhance the transfection efficiency [46]. The “cone” shape character of cationic lipids can be increased by their dimerization. Cullis et al [47] utilized this strategy to synthesize a novel dimer cationic lipid and achieved improved transfection activity as compared with a monomer lipid. Chien et al [48] adopted similar strategy to design novel cationic cardiolipin (dimeric cationic lipid) analogues. Cardiolipin has two negatively charged phosphate groups, which were replaced with quaternary ammonium groups termed CCLA. The c-raf-siRNA-CCLA complexes showed higher efficiency in comparison to DOTAP-based liposomes *in vitro* and in a SCID mouse model bearing human breast xenografts.

Although electrostatic association(s) of siRNAs with the chosen cationic lipid carrier is a critical element to generate suitable siRNA lipoplexes, helper lipids also play an important role in contributing their stability and delivery efficiency [49, 50]. Helper lipids with cone-shaped geometry such as dioleoyl phosphatidylethanolamine (DOPE), favor the formation of non-bilayer hexagonal _II_ phase to facilitate fusion with the endosomal membranes, resulting in their destabilization for efficient intracellular siRNA delivery [51]. However, the high fusogenic nature of DOPE renders less colloidal stability due to bilayer fusion during storage and increased serum protein interactions [52]. Cylindrical-shaped lipid phosphatidylcholines such as DOPC and DSPC favor the formation of a bilayer phase [53] and hence can provide higher stability to the bilayer for *in vivo* applications [49]. Cholesterol is often included as a helper lipid to provide stability to liposomes since its small size can fit into spaces between phospholipids [52]. However, the above mentioned lipids could not protect the liposomal formulations from opsonization and clearance due to the reticulo-endothelial system (RES) *in vivo*. Inclusion of a PEGylated helper lipid not only reduces particle size and enhances the liposomal colloidal stability but also increases resistance to opsonization and the RES. Methoxypolyethyleneglycol distearoyl phosphatidylethanolamine (mPEG-DSPE) is the most commonly used PEGylating lipid [49, 54]. It is important to note that although PEGylation improves circulation time *in vivo*, it reduces cellular internalization of lipoplexes which ultimately hinders the potential of siRNAs. To overcome these limitations researchers have incorporated weak linkers or cleavable linkers to PEGylated lipids that are sensitive to the cancer cell environment. This approach reduces the PEG density of the lipoplexes once in circulation thus promoting cellular interactions leading to enhanced siRNA delivery. PEGylated lipids modulate the stability and siRNA delivery efficiency of cationic liposomes. It is very important to note that the ratio of these lipids with respect to cationic lipid and with each other is very important. The imbalance of this ratio invites instability of the vector which in turn causes inefficacious delivery of siRNAs [49].

Several examples of lipid based vectors containing cationic lipids have been cited in this review; however, it is worth citing that using DOPC neutral liposomes, Sood and colleagues [55] reported studies for siRNA delivery to down regulate oncoprotein EphA2, which is over-expressed in bladder cancer.

It is encouraging to note that a number of lipid based siRNA formulations (examples, AtuPLEX [36] and Stable nucleic acid-lipid particles (SNALPs) [56, 57] ) are under clinical trials (Figure 2A). Below we provide details about two relatively new lipids, bolaamphiphiles and oxime ethers, which have shown promising RNAi activity in cancer cells.

#### 2.2.1 Bola lipids

The lipid systems described in the preceding sections are either focused on glycerol-based or non-glycerol backbone structures and bear one hydrophilic head group that is instrumental for interactions with siRNAs. However, it is known that glycerol-based lipids are likely to be degraded by phospholipases and hence the stability of lipoplexes gets compromised. These limitations prompted investigators to consider alternate lipid molecules as siRNA delivery platforms. Bolaamphiphiles belong to one such class of amphiphilic molecules that contain two hydrophilic groups linked with a hydrophobic skeleton with desired variations. In general, the hydrophilic groups of Bolaamphiphiles can be symmetric or asymmetric, and either non-ionic or ionic in nature. One of the most natural Bolaamphiphiles, tetra-ether lipids (bola lipids) that originate from lower organisms such as archaebacteria are known to be resistant to extreme conditions such as pH and temperature. In contrast to glycerol-based lipids, bola lipids self-assemble into monolayers with their positively charged head groups presented towards the exterior, available for interaction with negatively charged molecules such as siRNAs. The chemical structure of bola lipids studied in our laboratory are shown in Figure 2B. The first two lipids, GLH-19 and −20 contain an acetylcholine head group; however, GLH-19 contains a non-hydrolysable acetylcholine head group whereas GLH-20 contains a hydrolysable acetylcholine head group. Vesicles derived from these lipids were previously examined for their ability to cross the blood brain barrier (BBB) and regulate the release of their vesicle-entrapped contents [58].

**Figure 3.**
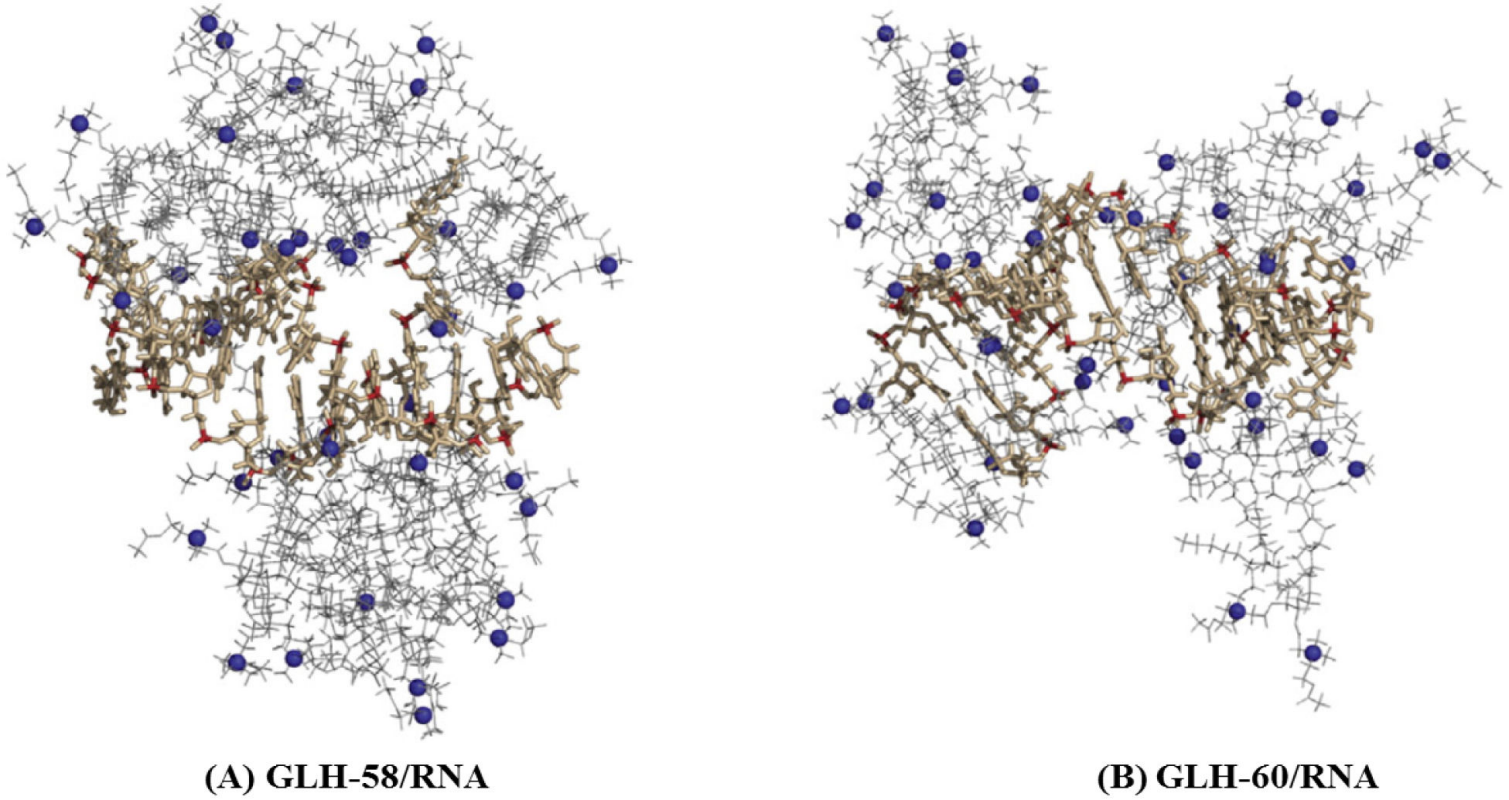
Molecular dynamics simulations for complex formation of bolas with RNA, (A) Bola GLH-58 with RNA and (B) Bola GLH-60 with RNA; bola hydrophobic skeleton (gray) its head groups (blue spheres). RNA (tan) its P atoms of the backbone phosphate groups (red) [59].

Our group examined a series of chemically defined bola lipids for their ability to form complexes with siRNAs and to deliver them for silencing genes [59, 60]. Bolas GLH-19 and GLH-20 have been shown to interact with RNAs *in silico* and were experimentally demonstrated to efficiently silence the GFP gene in human breast cancer cells [59]. Inspired by the promising data of GLH-19 and −20 as delivery agents for RNAs, recently we have explored two new bola lipids GLH-58 and GLH-60 (Figure 2B). These lipids were designed to examine the effect(s) of lipid head group positive charge(s) for higher gene silencing efficiency, by increasing the number of positive charges from 2 (GLH-58) to 4 (GLH-60) [60]. As expected, computationally (Figure 3) as well as biophysically it was observed that due to the higher number of positive charges, GLH-60 more tightly bound nucleic acid duplexes in comparison to GLH-58. This higher binding resulted in a dose-dependent increase in cellular uptake of nucleic acid duplexes compared to GLH-58 as expected. However, GLH-60 could not produce efficient GFP silencing at a given dose (5 μg/mL) presumably due to the insufficient release of dicer substrates of siRNAs (DsiRNAs). On the other hand, due to the relatively lower binding affinity of GLH-58, a sufficient amount of DsiRNAs were available to silence the GFP expression [60]. However, these observations warrant further investigation.

#### 2.2.2 Oxime ether lipids

Oxime ether lipids (OELs) are a relatively new class of cationic agents explored as efficient transfection tools [61, 62]. In contrast to commonly used transfection lipids, OELs contain oxime ether bonds (Figure 2C) and can be synthesized by simple and efficient click chemistry [63]. Oxime linkages are relatively stable at neutral pH, but cleavable at low pH values, thus providing a built-in nucleic acid release mechanism [64]. By synthesizing a variety of oxime ether lipids we demonstrated that hydrophobicity and the degree of unsaturation of the fatty acyl chains play a role in siRNA activity. Moreover, the head group polarities perform a role in the electrostatic interaction with siRNAs, protection from nucleases, uptake, and gene silencing in a cell culture system [62]. The structures of symmetric OELs, containing either saturated or unsaturated hydrophobic fatty acyl chains are shown in Figure 2C. We also introduced hydrophilic hydroxyl groups in the polar region of these molecules (Figure 2C). These lipids, along with equimolar concentration of DOPE, retained their ability to form vesicles as determined by cryo-electron microscopy studies. OELs were also found to be relatively non-toxic as determined by *in vitro* cell culture assays [62]. Interestingly, OELs that contain unsaturated bonds in the fatty acyl chains (Figure 2C, iii & v) demonstrated poor binding with nucleic acid duplexes in comparison to saturated lipids. However, introduction of OH-groups (Figure 2C, v) rescued its binding affinity to nucleic acids presumably due to favored electrostatic and /or polar interactions. Our results showed that the lipids containing unsaturated and hydroxylated head groups are better transfecting and silencing agents than the saturated and non-hydroxylated lipids [62]. Further detailed studies will validate the application of OELs in broader prospects for RNAi therapy.

### 2.3 Cationic polymers

**Figure 4.**
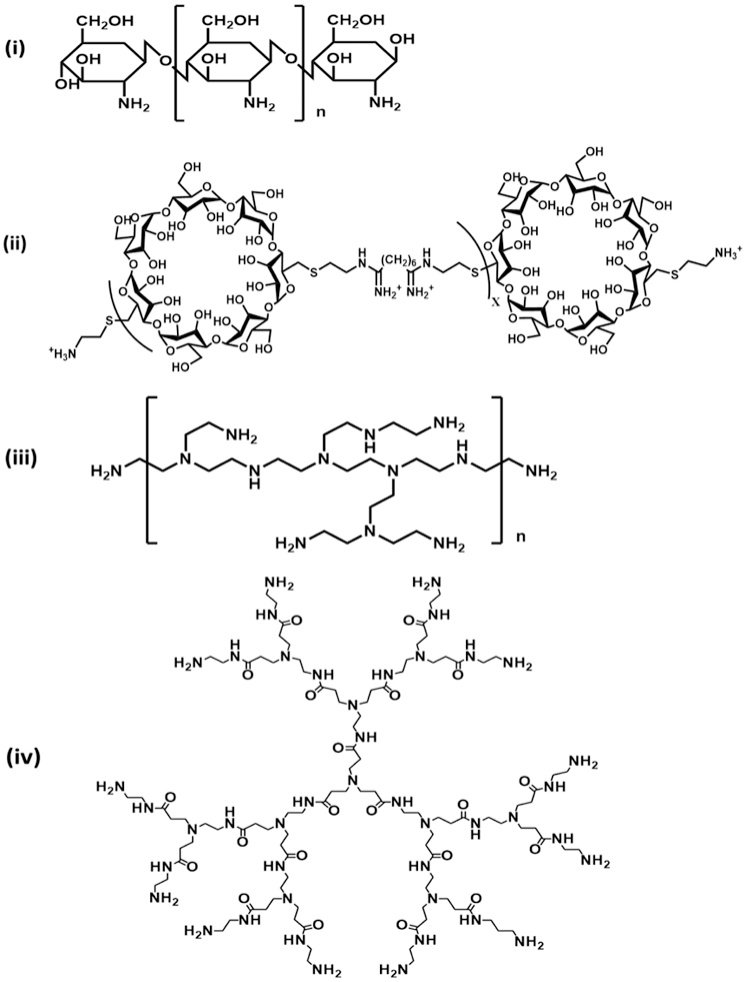
Chemical structures of cationic polymers. (A) Chitosan (CS), (B) Cyclodextrin containing polymers (CDPs), (C) Branched polyethyleneimine (bPEI), (D) Poly(amidoamine) dendrimers (PAMAM).

Polycation-based polymers are another class of materials that have been extensively exploited for the delivery of nucleic acids including siRNAs. Cationic polymers, upon electrostatic interactions with the phosphate-bearing anionic nucleic acids, form polyplexes and constitute a platform for their delivery [65, 66]. In 1965 Vaheri and Pagano showed that cationic diethylaminoethyl (DEAE) dextran could improve the transfection of phenol-extracted purified poliovirus RNA [67]. Since then several polymers have been designed and studied containing variations in their architectures, charge densities, and molecular weights. The designed polymers showed long persistence in blood and are biodegradable and biocompatible. In addition, inclusion of endosmolytic membrane agents and targeting ligands have also been investigated to improve the efficiency of the polymers for siRNA delivery. In this section, we will elaborate on the most extensively studied cationic polymers such as chitosan, cyclodextrin containing polycations, polyethyleneimine and poly(amidoamine) dendrimers for the delivery of siRNAs (Figure 4).

#### 2.3.1 Chitosan

Chitosan (CS) is a linear polysaccharide derived from deacetylation of chitin. It is comprised of arbitrarily disseminated N-acetyl-D-glucosamine and D-glucosamine units. Protonation of these glucosamine units mediate electrostatic interactions with anionic nucleic acids such as siRNAs. Chitosan is biodegradable, its high dose is well tolerated *in vivo*, and is rendered safe to use in clinical applications [68].

Although un-modified chitosan has shown promising results in siRNA delivery, these studies were limited only to particular cell models and experimental conditions. Interestingly, when they were subjected to different cell models and different experimental conditions a greater degree of variability in results was observed [68].

Chitosan’s amine and hydroxyl functional groups can be readily modified and functionalized to impart new properties to the polymer for efficient RNAi. However, the molecular weight, deacetylation degree of chitosan, formulation strategy with siRNAs and incorporation of membrane permeating, endosomolytic and targeting ligands also play a significant role in efficient gene silencing [68].

#### 2.3.2 Cyclodextrin containing polycations (CDPs)

Cyclodextrins (CDs) are enzymatically modified starch derivatives made up of D-glucopyranose units connected through α-(1-4) linkages with a donut shape or a truncated cone. According to the number of included glucose units they are designated by a Greek letter; α-,β- and γ-CDs consist of 6, 7 and 8 glucose units respectively [69]. CDs are generally modified with short cationic polymers such as polyamidine etc. to electrostatically complex negatively charged siRNAs to obtain cyclodextrin containing polycations [70].

#### 2.3.3 Polyethylenimine

The polycationic polymer polyethylenimine (PEI) is the most extensively studied polymer for nucleic acid delivery including siRNAs [71]. Protonated amino groups of PEI condenses siRNAs to form self-assembled nanoparticles. Delivery potential of PEI depends on its molecular weight, branching, and its inherent proton buffering capacity to escape endosomes via the “proton sponge” mechanism [72]. Despite its high transfection efficiency PEI is known to be toxic. It induces complement activation, triggers coagulation, permeabilizes plasma or mitochondrial membranes and on top of all this it is non-biodegradable [73]. To address such concerns, various degradable PEIs with low cytotoxicity have been synthesized by using a variety of cross-linking or grafting reagents [74]. Researchers have attached short oligoamine linkers onto long chains of polysaccharides (e.g. dextran, pullulan, chitosan, hyaluronic acid, alginic acid, gellan gum, cyclodextrins, etc.), which usually act as a template to tether these oligoamines via a degradable linkage, i.e. esters, disulfides, carbamates, amides, to make these grafted polymers non-toxic compared to high molecular weight branched polyethyleneimine (bPEI, 25 kDa), which is considered as a “gold standard” in gene delivery applications [75] .

#### 2.3.4 Poly (amidoamine) (PAMAM) dendrimers

The word “dendrimer” (derived from the Greek word “dendron” meaning tree and “meros” meaning part), portrays a structure that comprises a central core molecule acting as a root, as well as reactive end groups to allow the addition of repetitive units or branching in a symmetrical controllable manner [76]. Moreover, modification of the end groups enable conjugation with various ligands to present multiple copies of therapeutics and other agents for biomedical applications [77, 78].

Due to their unique and adaptable chemistry, various kinds of dendrimers have been explored for siRNA delivery, including poly(amidoamine) dendrimers, poly(propyleneimine) dendrimers, carbosilane dendrimers, poly(L-lysine) dendrimers, triazine dendrimers, polyglycerol based dendrimers, nanocarbon-based dendrimers, and other types of dendrimers [76]. But in this section we are focusing on the extensively studied poly(amidoamine) dendrimers (PAMAM) dendrimers.

Several generations of PAMAM dendrimers were initially exploited owing to their inherent cationicity to condense siRNAs, binding ability, protection of bound siRNAs against nucleases and subsequently their delivery to cancer cells without any modification to their native structure. Since these dendrimers are cationic they have inbuilt capability to facilitate endosomal escape of PAMAM/siRNA complexes via the proton sponge effect like other polycationic polymers.

## 3 Functionalization of NVCVs for siRNA delivery

The essentials of a successful siRNA delivery system include (i) protection of siRNAs from nuclease mediated degradation during systemic circulation; (ii) promotion of enhanced cellular uptake; (iii) endosomal escape; (iv) target site accumulation while evading nonspecific uptake in normal and non-target tissue; and (v) empowering release of siRNAs in the cytosol [6, 15, 79, 80]. Conventional NVCVs, following complexation with the siRNAs, provide protection against nucleases and also aid in delivering siRNAs across the cell membranes; however, these NVCVs/siRNA complexes failed to produce remarkable gene silencing. Therefore, researchers have developed functionalized NVCVs that provide an edge over conventional counterparts with the components that facilitate improved siRNA delivery [81-84]. In the below section, we will discuss selected functionalization strategies that when singly incorporated in NVCVs showed better performance in siRNA delivery than their non-functionalized counterparts.

### 3.1 Incorporation of lipids

#### 3.1.1 CPPs conjugated with lipids

CPPs alone can mediate cellular entry of siRNAs; however; the high CPP to siRNA ratios required to translocate across cell membranes result in non-specific cytotoxicity in cells. Several reports showed that lipid-peptide conjugates are viable systems for nucleic acid delivery [85, 86] as lipid or fatty acid conjugation to peptide increase the hydrophobicity of the CPPs to facilitate interaction with the cell membranes.

In a study by Kim and colleagues [81] conjugation of cholesterol (a lipid) to oligo-d-arginine (Chol-R9) and the ability to deliver vascular endothelial growth factor (VEGF)-siRNA to cancer cells was investigated. VEGF is a multifunctional angiogenic growth factor that is involved in the development and maintenance of a vascular network in the vascularization of solid tumors. Chol-R9 significantly reduced the VEGF gene expression almost by half in comparison to unmodified R9 in CT-26 (human colon adenocarcinoma). Moreover, in a mouse model bearing a subcutaneous tumor, the local administration of complexed VEGF-siRNA significantly regressed the tumor in comparison to controls.

#### 3.1.2 Cationic polymers grafted with lipids

##### 3.1.2.1 PAMAM dendrimers with lipids

A small amphiphilic dendrimer comprising of hydrophobic alkyl chain-lipid and lower generation hydrophilic PAMAM dendron that exploited properties of both lipid and polymer to form stable complexes with siRNAs could induce potent gene silencing and cause anticancer effects *in vitro* and *in vivo*. Among the lipid/PAMAM systems synthesized triethanolamine (TEA)-PAMAM dendrimer functionalized with heptadecanoic azide, demonstrated superior silencing over TEA-PAMAM at the concentration of 50 nM. This delivery vector efficiently delivered GL3Luc-siRNA (for luciferase) in A549 Luciferase cells and HSF1-siRNA (for heat shock factor 1) and Hsp27-siRNA (for heat shock protein 27) in PC-3 cells. In fact, the lipid/PAMAM mediated silencing by Hsp27-siRNA was maintained even after 7 days in PC-3 (prostate cancer) xenografted nude mice [83].

### 3.2 Inclusion of endosome rupturing or protonable agents

After being endocytosed into the cells NVCVs/siRNA complexes face the problem of endosomal escape failure resulting in poor RNAi efficiency. Incorporation of endosomolytic or protonable agents to NVCVs facilitated endosomal escape and improved siRNA delivery (See Table 1). The following are a few examples.

#### 3.2.1 CPPs and endosomolytic agents

RNAi mediated by CPPs can be augmented by directly fusing CPPs with the endosmolytic peptides. HA2 peptides derived from hemagglutinin HA2 subunit, under acidic (pH~5.5) conditions of endosomes gets protonated and disintegrates the bilayer of endosomes upon fusion. It was observed that HA2 fusion peptides, when linked to penetratin, enhanced luciferase-siRNA delivery efficiency in HeLa (human cervix adenocarcinoma) and HepG2 (hepatocellular carcinoma) cells [87]. Similarly, R9 peptide fused with an endosomal membrane disruptive peptide (INF-7) derived from influenza virus delivered 18 fold more siRNAs to CAL27 and SCC-25 human squamous oral cancer cells in comparison to controls. siRNAs targeted to cancerous inhibitor of protein phosphatase 2A (CIP2A) delivered by this chimeric peptide significantly resulted in decreased oral cancer cell invasiveness and reduction in cancer metastasis [26].

Another study focused on conjugating histidine’s imidazole group to CPPs since its pKa is close to the pH in endosomes. The resulting complexes were predicted to induce endosomal escape via the ‘proton sponge effect’ subsequent to endosomal membrane rupture. Incorporation of five histidine’s to dermaseptin derived CPP, S4_13_-PV showed effective gene silencing of survivin gene in HT1080 (human fibrosarcoma) cells [88].

#### 3.2.2 Cationic lipids and endosomolytic agents

Researchers have been incorporating agents or synthesizing lipids that are protonable under the acidic environment of endosomes for improving liposomal mediated siRNA delivery. The protonable imidazole ring of histidine and polymer polyethyleneimine has been widely used to design endosomal pH sensitive vectors [89]. In this regard, one such example where imidazole/imidazolium lipophosphoramidate and histidinylated polyethyleneimine were studied for the formulation of siRNA lipopolyplexes (LPRi) to efficiently deliver enhanced green fluorescent protein (EGFP)-siRNA and luciferase (Luc)-siRNA in HeLa or B16F10 (mouse melanoma) cells stably expressing EGFP or luciferase respectively. The N-methylimidazolium lipophosphoramidate sports a permanent positive charge to interact with negatively charged siRNAs whilst the imidazole moiety of the histamine lipophosphoramidate and histidinylated polylysine acquires a positive charge below pH 6. The higher gene silencing activity of LPRi, in comparison to its control counterparts and commercial transfection agents including JetPRIME™, could be attributed to the inclusion of these protonable groups that can induce an acid-mediated endosomal membrane destabilization by augmenting the fusogenic properties between the endosomal membrane and the cationic imidazole-based lipid and/or by a proton sponge effect [82].

Stable nucleic acid-lipid particles (SNALPs), originally described by Heyes et al [56] & Morrissey et al [57] are being considered as one of the most promising particles for RNAi therapy. These particles contain ionizable cationic lipid, 1,2-dilinoleyloxy-3-dimethylaminopropane (DLinDMA, Figure 2A) along with helper lipids and a PEG-lipid. DLinDMA is an ionizable cationic lipid with pKa_s_ < pH 7.0 that can complex siRNAs at pH 4 and maintain a neutral or low cationic surface charge density at pH 7.4. Since DLinDMA undergo protonation at low pH it renders the liposomal formulation amenable to efficient intracellular uptake without forfeiting the endosomal release potential. DLinDMA liposomal formulations were tested to deliver cell cycle regulating proteins, polo-like kinase (PLK1) and kinesin spindle protein (KSP) to hepatic cancer model (Hep3B, Neuro2a hepatic tumor) [90]. Inhibition of PLK1 and KSP caused cell-cycle arrest and induction of apoptosis in cancer cells. Liposomal mediated delivery of PLK1-siRNA and KSP-siRNA significantly inhibited the PLK1-mRNA and KSP-mRNA expression levels in hepatic cancer cell model and thus enhanced the survival rate of tumor bearing mice.

#### 3.2.3 Catioinic polymers and endosmolytic agents

##### 3.2.3.1 Chitosan

Modification of chitosan with endosome-rupture agents facilitated the release of siRNAs for efficient silencing due to protonation of their amino groups at acidic endosomal pH. PEI, poly-L-arginine (PLR) and cationic CPPs, due to their high buffering capacity at acidic endosomal pH, can act as endosomal disrupters when either grafted on to the chitosan or mixed with it. Addition of these influenced solubility, stability and size of the overall polyplex which in turn enhanced the transfection and silencing efficiency [91, 92].

Chitosan grafted bPEI on complexation with GFP-siRNAs knocked down the GFP expression in human lung carcinoma A549 cells by 2.5 times in comparison to the PEI/siRNA complexes. Another gene Akt1 involved in lung tumorigenesis and cell proliferation was also targeted by the same formulation of chitosan-bPEI. Akt1-siRNA treatment showed significant increase in apoptosis and reduced cell proliferation of A549 cells [91].

In an another study, where PLR was incorporated to a PEGylated formulation of chitosan (PEG-chitosan-PLR) not only caused significant reduction in the expression of survivin via survivin-siRNA in Hepa 1–6 (mouse hepatoma), A549 (human lung carcinoma), and VK2 (human vagina endometriosis) cells and of GFP in 293-T (human embryonic kidney) GFP cells but also stands high serum tolerance (upto 50%). Chitosan-PLR conjugates showed similar levels of target gene expression reduction but the formulation was not compatible with increased serum levels [92].

##### 3.2.3.2 Cyclodextrin containing polycations

A star-shaped CDP, β-CD-g-P(HMA_79_-co-DMAEMA_33_-co-TMAEMA_48_)_4.8_ polymer that includes pH-sensitive 2-(dimethylamino) ethyl methacrylate (DMAEMA) monomers linked to CD acid-labile hydrazone linkages was developed to facilitate endosomal escape for siRNAs delivery. Cationic TMAEMA was incorporated to provide electrostatic interaction with siRNAs. Overexpression of RhoC-GTPases (RhoC) was considered as a therapeutic target for inhibiting breast cancer metastasis. These CDP/RhoC-siRNA complexes together form “smart” particles at a low N/P ratio of 2.5/1 which successfully escaped the endosomes due to DMAEMA, and almost completely suppressed the RhoC protein levels in SUM149 and MDA-MB-231 breast cancer cells. Moreover, these particles retard the invasion, motility, and migration of these cells [93].

### 3.3 Incorporation of bioreducible linkages

After endososmal escape of NVCV/siRNA complexes, siRNAs should be delivered free in the cytoplasm to reach the RISC assembly. Glutathione (GSH) is known to reduce disulfide linkages. The concentration of glutathione is estimated to be up to 1000-fold higher in tumor cells than in the blood and the extracellular levels of GSH in a tumor mass are 100-fold higher than in normal tissues [94]. The strategy of including glutathione reducible disulfide linkages in NVCVs has been exploited to improve siRNA delivery in cancer cells. Here are few examples.

#### 3.3.1 CPPs and bioreducible linkages

In this context, Won et al [95] synthesized bioreducible poly (oligo-d-arginine) (rPOA) with two cysteines Cys-9DR-Cys and demonstrated the fast decondensation of poly(oligo-d-arginine)/VEGF-siRNA polyplexes in the cytoplasm in response to intracellular glutathione (GSH) in SCC (human squamous cell carcinoma) cells *in vitro* to reduce the VEGF induced secretions, mRNA levels, and angiogenesis in cancer cells. VEGF-siRNA delivered by rPOA significantly reduced VEGF secretions and VEGF mRNA levels *in vitro* in comparison to bPEI and PLR. Moreover, tumor growth was suppressed *in vivo* by VEGF-siRNA/rPOA in subcutaneous SCC animal model.

#### 3.3.2 Cationic polymers and bioreducible linkages

##### 3.3.2.1 Polyethyleneimine and bioreducible linkages

Synthesis of degradable PEIs with disulfide linkages in the side chain of the polymer based on low molecular weight (2600, 3100 and 4600 Da) PEI and N,N′-bis (terbutoxycarbonyl) cysteine or 3,3′-dithiodipropionicacid di (N-succinimidyl ester) as cross-linkers has been reported [96]. The polymers showed high transfection efficiency with low toxicity when compared to commercial transfection reagents. The results further showed that the combination of high branching density and reductively cleavable bonds in the polymer is a very important parameter for efficient gene silencing by siRNAs [97].

Kumar and co-workers [98] prepared bioreducible PEI nanoparticles via crosslinking bioreducible linker, 3,3’-dithiodipropionic acid (DTPA) to bPEI to form DP nanoparticles. DTPA blocks the charge density of bPEI and its crosslinking is converted it to NPs. Originally, the NPs were used for the transfection of pDNA where it showed high transfection efficiency. The versatility of the DP vectors was proved by the sequential delivery of GFP-siRNA into MCF-7 breast cancer cells. DP-siRNA nanoparticles effectively silenced the expression of GFP. The higher transfection and silencing efficiency could be attributed to the glutathione-mediated reduction of disulfide bonds.

##### 3.3.2.2 PAMAM dendrimers and bioreducible linkages

Recently, the group of Sung Wan Kim [99] demonstrated that the disulfide bonds in bioreducible poly(cystaminebisacrylamide-diaminohexane) (ABP) can be cleaved by cellular reducing agent glutathione (GSH). Arginine grafted bio-reducible ABP was incorporated to PAMAM dendrimers to deliver anti-vascular endothelial growth factor (VEGF)-siRNA to cancer cell lines such as human hepatocarcinoma (Huh-7), human lung adenocarcinoma (A549), and human fibrosarcoma (HT1080) cells. VEGF is responsible for vasculogenesis and angiogenesis in cancer. Delivery of VEGF-siRNA silences the vasculogenesis and angiogenesis in cancer. PAMAM-ABP dendrimers significantly reduced VEGF expressions in Huh-7, A549, and HT1080 cells in comparison to PEI/VEGF-siRNA complexes.

### 3.4 Incorporation of CPPs

Since CPPs have the inherent ability to traverse across cell membranes, researchers have been using them along with other delivery vectors such as lipid and polymer based vectors for improving siRNA delivery [84, 100]. CPP associated delivery vectors of lipids and polymers are discussed below.

#### 3.4.1 Cationic lipids and CPPs

It was shown that CPPs employed with cationic liposomes imparted better penetration capability to liposomes delivering nucleic acids across cells [100]. Zhang et al [84] formulated DOTAP based formulation surface modified with oligoarginine (R8) for the delivery of human double minute gene 2 (HDM2)-siRNA. HDM2 gene is involved in cell proliferation. R8-liposomes provided very high transfection efficiency in all three types of lung cancer cells (lung squamous cell carcinoma (SK-MES-1), non-small cell lung carcinoma (A549), and small cell lung carcinoma (NCI-H446)) tested even in the presence of serum with very low cytotoxicity. This R8 liposomal HDM2-siRNA formulation demonstrated significant lung cancer growth inhibition with respect to lipofectamine 2000, mock DOTAP liposomes, and siRNAs alone.

#### 3.4.2 Cationic polymers

##### 3.4.2.1 Chitosan

TAT peptide is one of the excellent CPPs that have been used to deliver a variety of cargoes including siRNAs. Gao and coworkers directly grafted TAT peptides to the primary amino groups of the chitosan molecules to obtain a co-polymer of TAT-g-CS as a non-viral vector for siRNA delivery [101]. Significant reduction in the luciferase reporter gene in MCF7-Luc cells showed the capability of TAT-g-CS to deliver siRNAs. The silencing efficiency mediated by TAT-g-CS/Luc-siRNA complexes was 3.7 folds higher than the CS/ Luc-siRNA complexes. Further, TAT-g-CS was also employed to evaluate the anti-proliferation potential of survivin-siRNA for anti-cancer therapy. TAT-g-CS successfully delivered survivin-siRNA to 4T1 luciferase cells as indicated by the high rate of apoptosis and inhibitory potential on tumor metastasis in 4T1 cells inoculated metastatic breast cancer tumor model in mice [101].

In another study, [102] nonaarginine (R9), a CPP, was conjugated to the chitosan NPs to enhance the intracellular delivery of siRNAs. R9-CS NPs delivered CypB-siRNA targeted to cyclophillin B protein efficiently in HeLa cancer cells. The R9-CS NPs showed a higher degree of knockdown of the cyclophillin B protein in comparison to the unmodified CS NPs.

### 3.5 Incorporation of targeting ligands

Specific targeting to cancer cells without affecting the normal cells is one of the major problems faced by the NVCVs. To address this, researchers are exploiting the advantage of coupling cell targeting ligands to the NVCVs. Active cell targeting is a promising approach that has the potential for precluding the unwanted entry of delivery systems to cells other than the target cells. Conjugation of a particular cell receptor or biomarker specific ligand to the surface of NVCVs would enable cell specific delivery of siRNAs [79, 103]. Herein, we are discussing a few examples of NVCVs that when coupled with targeting ligands showed selective siRNA delivery to cancer cells (See Table 2).

#### 3.5.1 CPPs and targeting ligands

In a recent study [104], WFLLTM (A1), a 6 amino acid peptide with high selectivity for vascular endothelial growth factor receptor-1 (VEGFR1) overexpressed on almost all tumor cells, was conjugated to TAT to form a tumor-selective CPP. TAT-A1 selectively penetrated into tumor cells when added to co-cultured tumor cells and normal cells due to the recognition of VEGFR1 receptors. Glyceraldehyde 3-phosphate dehydrogenase (GADPH) gene involved in the catalysis of glycolysis to break down glucose for energy and carbon molecules was chosen as a target for evaluating the TAT-A1 capability to deliver GAPDH-siRNA. The silencing effect of GAPDH-siRNA transferred by TAT-A1 was observed at both mRNA and protein levels in HepG2 cells. Significantly lower GAPDH mRNA expression was observed for TAT-A1 than Lipofectamine 2000. A similar silencing effect of GAPDH at both the mRNA and protein levels was noted in other cells also including HT29 (human colorectal adenocarcinoma), HL60 (human promyelocytic leukemia), L02 (normal hepatocytes) and HUVEC (human umbilical vein endothelial cells) cells.

#### 3.5.2 Cationic lipid based vectors and targeting ligands

Folate receptors are overexpressed in many cancers including lung cancer. In addition to this, many human cancers including lung cancer overexpress an RNA binding protein Human antigen R (HuR), which is responsible for regulating the expression of several oncoproteins [105]. Ramesh and coworkers [106] investigated the efficacy of folate receptor-α (FRA)-targeted DOTAP: Cholesterol lipid nanoparticles encapsulated with HuR-siRNA (HuR-FNP) against H1299 lung cancer cell line and the results were compared to normal lung fibroblast (CCD16) cells with low to no FRA expression. It was hypothesized that siRNA mediated silencing of HuR will cause universal knockdown of oncoproteins resulting in decreased survival of cancer cells. It was shown that HuR-FNP was selectively taken up by FRA overexpressing lung cancer cells. This liposomal formulation significantly inhibited the HuR gene resulting in reduced expression of oncoproteins, cell cycle arrest, tumor cell proliferation, and tumor cell migration.

#### 3.5.3 Cationic polymer based vectors and targeting ligands

##### 3.5.3.1 Chitosan and targeting ligands

Folate receptors are overexpressed in malignant cells such as HeLa and human ovarian adenocarcinoma (OV-3) cells [107]. Qin Shi group utilized low molecular weight chitosan of 25 and 50 kDa grafted with folate ligand to deliver sjogren syndrome antigen (SSB)-targeted siRNA to the above cancer cells. It was observed that the 25 kDa or 50 kDa folate-chitosan-siRNA showed significant knockdown of SSB mRNA expression in HeLa cells compared with 25 kDa and 50 kDa non folate chitosan-siRNA formulations. Moreover, in OV-3 cells greater inhibition of SSB gene expression was observed with 50 kDa folate-chitosan-SSB siRNA in comparison to 25 kDa folate-chitosan-SSB siRNA. It is important to note that no SSB mRNA inhibitory effect was observed with the similar chitosan/SSB-siRNA complexes in folate receptor-negative MG-63 (human osteosarcoma) cells [107].

In an interesting study, Corbet et al [108] showed that targeting tumor metabolism using siRNAs contribute to an attractive therapeutic strategy for treating cancer. Lactate transporter MCT1 and the glutamine transporter ASCT2 are the two key transporters of energy fuels for cancer cells. Human non-small cell lung carcinoma (H1299) and human cervix squamous cell carcinoma (SiHa) cells overexpress these transporters. In addition to these transporters *αvβ*_3_ integrin receptors are also over-expressed on these cells. Non-covalently PEGylated chitosan based nanoparticles linked with RGDp peptidomimetic to target *αvβ*_3_ integrin receptors specifically deliver MCT1 and ASCT2 siRNAs to silence the lactate transporter MCT1 and the glutamine transporter ASCT2 expression levels in H1299 and SiHa cells. The strategy led to the down-regulation of tumor metabolism transporters which in turn pushed dramatic tumor growth inhibition in mice.

##### 3.5.3.2 Cyclodextrin containing polycations and targeting ligands

Davis group for the first time synthesized linear cationic β-CD containing polymers [109]. The group showed that the CDP polyplexes could efficiently deliver luceriferase targeted siRNAs (Luc-siRNAs) to Neuro 2A tumor xenografts in mice. These CDPs were incorporated with adamantane–PEG (AD–PEG) and adamantane–PEG–transferrin (AD–PEG–Tf) to increase the circulation time and targeting to CD71 transferrin receptor respectively. CDPs were also modified with imidazole groups to escape endosomes. These nanocomplexes, named as CALAA-01, were the first polymer system entered into clinical trial for the delivery of siRNAs. Calando Pharmaceuticals was successful in taking these CDP/siRNA nanosystems to be the first in-human phase-I clinical trial that involved targeted systemic administration of siRNA to solid tumors. Davis group studies revealed that targeting is important for the specific delivery of siRNAs to the diseased cells [109]. However, further phase clinical trials were stopped due to the drug instability which was supposedly thought to be due to the presence of the transferrin ligand [110].

Recently, Fitzgerald et al [111] reported the first example of using an anisamide ligand that targets the sigma-1 receptor over-expressed on prostate cancer cells for the delivery of siRNAs via cationic β-cyclodextrin PEG-adamantane nanoformulation. Like in other cancers, Polo-like kinase 1 (PLK1), a serine-threonine-protein kinase enzyme, is overexpressed in prostate cancer. It plays an important part in cellular mitosis and high levels are connected with amplified cell proliferation [112]. This nanoformulation demonstrated significant cellular uptake of siRNAs and corresponding PLK1 gene knockdown in prostate cancer cells (DU145, VCaP and PC3). The nanoformulation protected the siRNAs from degradation and was non-toxic [111].

##### 3.5.3.3 Polyethyleneimine and targeting ligands

An amphiphilic and biodegradable ternary copolymer, polyethylenimine-graft-polycaprolactone-block-poly-(ethyleneglycol)-folate (PEI-PCL-PEG-Fol) was synthesized and demonstrated for targeted siRNA delivery via folate-FR (folate receptor) recognition. The biodegradability of the polymer was due to the hydrolysis of the PCL. Targeted delivery was observed in FR over-expressing SKOV-3 cells. PEI-PCL-PEG-Fol/siRNA polyplexes showed increased cellular uptake of fluorescent siRNA and *in vitro* gene knockdown of GAPDH-siRNA in SKOV-3 cells at an N/P ratio of 5 in comparison with the non-folate-conjugated micelleplexes and lipofectamine 2000. Authors claimed stronger inhibition of gene knockdown upon competition with free folic acid than others. The targeted recognition of folate, suitable size, good stability, and low cytotoxicity were the factors responsible for the greater knockdown activity of PEI-PCL-PEG-Fol. Moreover, the polymer/siRNA system showed enhanced circulation times and bioavailability and accumulated in subcutaneous ovarian cancer xenografts [113].

##### 3.5.3.4 PAMAM dendrimers and targeting ligands

Li et al. have reported a smart PAMAM-PEG dendrimer system conjugated with the cyclic tripeptide sequence arginine–glycine–aspartic acid (cRGD) for the delivery of human ether-à-go-go-related (hERG)-siRNAs in human anaplastic thyroid carcinoma (ATC HTC/3) cells. cRGD recognizes the α_v_β_3_ integrin receptor over-expressed in various cancer cells including ATC HTC/3cells. Overexpressed hERG amends the resting membrane potentials of cancerous cells to more depolarized values and repolarizes them to facilitate cell cycle progression which in turn provokes proliferation [114]. This multivalent smart formulation significantly reduced the expression of the hERG and induced cell death and apoptosis in HTC/3 cells. Moreover, downregulation of hERG resulted in the decrement of VEGF secretion and activation of caspase-3 [115].

## 4 Multifunctional NVCVs (with two or more functionalities)

As discussed above, incorporating selective individual functionalities in NVCVs did influence siRNA delivery efficacy but researchers have also tried incorporating various functionalities in single carriers to do different functions in a synergistic manner and to avoid unwanted side effects. Herein, below we will be discussing MNVCVs containing one NVCV chosen as the main component for siRNA delivery that will be further modified with two or more functionalities for enhancing siRNA delivery efficiency in comparison to the unmodified one.

### 4.1 Multifunctional CPP based vectors

To this view, Sangeeta Bhatia and co-workers [116] produced a library of 18 linear membrane-translocation domains that contained both polycationic peptide sequences [oligoarginines of varying net charge, TAT (48-60), and the HSV-1 tegument protein VP22] and amphipathic sequences (Penetratin (PEN) and Transportan (TP). Since lipid moieties such as myristoyl group are known to facilitate interactions with cell membranes [117], a myristoyl group (lipid portion) was added to the amino terminus to facilitate interactions with membrane lipids. These tandem peptides were made into targeting agents by linking to LyP-1 ligand that selectively target p32 receptors over-expressed at the surface of cancer or tumor cells such as HeLa , MDA-MB-435 (human melanoma) and OVCAR-8 (human ovarian adenocarcinoma). After binding to p32 receptors, LyP-1 was proteolytically digested by endogenous proteases to expose a cryptic CendR motif that triggered tissue penetration. It was observed that myristoylated tandem peptide, myr-TP-LyP-1, which condensed siRNAs into multivalent nanocomplexes, effectively delivered siRNAs in a cell-type dependent manner.

### 4.2 Multifunctional cationic lipid based vectors

Gujrati et al [118] recently synthesized a cationic lipid vector, (1-aminoethyl) iminobis [N-(oleicylcysteinyl-1-amino-ethyl)-propionamide]) (ECO) and assessed its siRNA delivery potential in silencing the luciferase gene in U87 glioblastoma cell line even in serum conditions. Since the ECO lipid is itself cationic, it can complex negatively charged siRNAs. In addition to this, ECO lipid bears the advantage of having a protonable ethylenediamine head group at endosomal pH and free thiols that autooxidize to disulfide linkages and stabilize ECO/Luc-siRNA complexes and get reduced back to thiols in the reducing cellular environment. Upon intracellular entry of complexes the pH sensitive nature of ECO promoted its endosomal escape. The released nanoparticles encountered cellular glutathione which reduced the disulfide bonds formed on the nanoparticles and facilitated the release of siRNA to cause robust gene silencing in U87-Luc cells at an optimal N/P ratio of 10.

### 4.3 Multifunctional cationic polymer based vectors

A multifunctional small molecule PEI (600 Da) based nanoparticle with octyl chain modification, bioreducible linkages and detachable cross-linked hyaluronic acid (HA) shell, HA-PSR, was developed to evaluate the anti-cancerous activity of polo-like kinase 1 (PLK1) specific siRNA (PLK1-siRNA) and Paclitaxel (PTX) in lung cancer model of A549 cells *in vitro* and *in vivo* [119]. PLK1 is involved in cell cycle regulation and is considered as proto-oncogene. Loss of PLK1 expression can induce pro-apoptotic pathways and inhibit growth in cancer cells. Whereas PTX is an anticancer small molecule hydrophobic drug that is involved in the breakdown of the microtubules during cell division. Octyl and PEI in the nanoparticle provided hydrophobic and cationic regions for encapsulation of PTX and PLK1-siRNA respectively. The thiolated hyaluronic acid (HA-SH) cross-linked outer shell in the nanoparticles not only provided selective delivery to CD44 receptors (glycoprotein involved in cell-cell interaction, adhesion, and migration) in overexpressing lung cancer cells but also offers protection from undesired drug release during circulation. The multifunctional nanoparticle encounters hyaluronidase (HAase) particularly in tumor microenvironments and de-shields HA from the inner core of the NPs, followed by GSH triggered reduction of the disulfide linkages in the cytoplasm to produce a co-burst release of both the cargos. Fast intracellular trafficking of HA-PSR nanoparticles maximized the synergistic cytotoxicity of PTX and PLK1-siRNA for remarkable tumor suppression.

## 5 Summary and Perspective

Since the discovery of RNAi it has been signaled as one of the best therapeutic interventions for the treatment of any type of disease including cancer by means of knocking down the genetic expression of the disease causing genes via RNAi inducers [6, 79, 80, 103]. However, the delivery of RNAi inducers including siRNAs is compromised due to their degradation by nucleases, high anionic charge, immunogenicity, and off target effects. To circumvent these issues non-viral cationic vectors (NVCVs) have emerged as one of the successful delivery vectors for the delivery of siRNAs. Although native or first generation NVCVs provide protection against nucleases and mask the negative charge of siRNAs, they suffer with the problem of insufficient uptake by cells, endosomal escape failure, and off-target induced cytotoxicity. Initially, investigators functionalized non-viral cationic vectors (NVCVs) that address only one issue at a time, (1) conjugation of lipids or CPPs to facilitate entry across cell membranes, (2) improved siRNA release via bioreducible linkages or pH sensitive linkers, and (3) receptor targeting for selective cellular delivery. Since these strategies were able to do only one function at a time, they encountered the problems of not having all the properties in a single system and ended up with issues that could only be fulfilled by other properties.

Multifunctional non-viral cationic vectors (MNVCVs) can be an answer for effective siRNA induced cancer therapy as they incorporate various distinct functions or properties within a single system where each individual component should function in harmony with the other components. MNVCVs armed with the assets of stability, biodegradability, biocompatibility, longer blood circulation, avoidance of the MPS system, non-immunogenicity, high loading capacity, target specific ligands, inhibitors of drug resistant machinery, internal or external stimuli responsive moieties and with the ability to release encapsulated entities such as siRNAs in the cytoplasm and anti-cancer drugs in a controlled manner can make a difference for siRNA therapy for cancer. This review discussed several examples of MNVCVs incorporated with different functionalities effectively delivered siRNAs.

MNVCVs may provide an edge over singly functionalized NVCVs in terms of simultaneously addressing various properties in one system. However, more complexity is being introduced to that system. As a result, there is a possibility of batch to batch variations which may be due to differences in either size, zeta potential, shape, chemistry, or surface properties that ultimately may cause altered biological activity with every batch tested. To avoid such unwarranted variations one should take appropriate amount/ratios of the material needed and also strictly replicate the experimental conditions while synthesizing MNVCVs which would otherwise lead to questionable results and toxic issues. This is important since the reproducibility and the non-toxicity is required to get permission from the FDA to take them to clinical trials.

In the end, we suggest that the road for efficient siRNA induced cancer therapy is on ride with MNVCVs equipped with different specific synergistically working functionalities, provided that these systems should specifically target cancer cells at lower doses and avoid healthy cells to preclude any unwanted side effects.

## Figures and Tables

**Table 1.** Incorporation of endosomolytic agents as functionalization in non-viral cationic vectors (NVCVs) for improved siRNA delivery.

NVCVs	Endosomolytic agents	Target gene	Cancer model	Ref
CPP-Penetratin	Hemaagglutinin HA2	Luciferase	HeLa, HepG2	[87]
CPP-Oligoarginine (9R)	INF-7	CIP2A	CAL27 and SCC-25	[26]
CPP-S4_13_-PV	Histidine	Survivin	HT1080	[88]
Cationic lipid-DOTAP	Histidinylated polyethyleneimine	EGFP, Luc	HeLa, B16F10	[82]
Cationic lipid-DLinDMA	DLinDMA	PLK1, KSP	Hep3B	[90]
Cationic polymer-Chitosan	Branched polyethyleneimine (bPEI)	Akt1	A549	[91]
	Poly-L-arginine (PLR)	Survivin	Hepa 1-6, A549 and VK2	[92]
Cationic polymer-CDPs	2-(dimethylamino) ethyl methyacrylate	Rho-C	SUM149, MDA-MB-231	[93]

**Table 2.** Incorporation of targeting ligands as functionalization in non-viral cationic vectors (NVCVs) for improved siRNA delivery.

NVCVs	Ligand	Target receptor	Target gene	Cancer model	Ref.
CPP-TAT	WFLLTM	VEGFR1	GAPDH	HepG2, HT29, HL60	[104]
Cationic lipid-DOTAP	Folic acid	Folate-α	HuR	H1299	[106]
Cationic polymer-Chitosan	Folic acid	Folate	SSB	HeLa, OV-3	[107]
	RGDp	αvβ_3_ integrin	MCT1, ASCT2	H1299	[108]
Cationic polymer-CDPs	Transferrin	CD71	Luc	Neuro2A	[110]
	Anisamide	Sigma-1	PLK1	DU145, VCaP, PC3	[111]
Cationic polymer-PEI	Folic acid	Folate	GADPH	SKOV-3	[113]
Cationic polymer-PAMAM	Lactobioic acid	Asialoglyco-protein	AEG1	HCC, QGY-7703	[120]
	cRGD	α_v_β_3_ integrin	hERG	human ATC HTC/3	[115]
